# Characterization and functional analysis of extrachromosomal circular DNA discovered from circulating extracellular vesicles in liver failure

**DOI:** 10.1002/ctm2.70059

**Published:** 2024-10-15

**Authors:** Yongbing Qian, Xiaoning Hong, Yang Yu, Cong Du, Jing Li, Jiaying Yu, Wenjun Xiao, Chen Chen, Defa Huang, Tianyu Zhong, Jiang Li, Xi Xiang, Zhigang Li

**Affiliations:** ^1^ Department of Liver Surgery Ren Ji Hospital Shanghai Jiao Tong University School of Medicine Shanghai China; ^2^ Scientific Research Center The Seventh Affiliated Hospital Sun Yat‐sen University Shenzhen China; ^3^ Cell‐gene Therapy Translational Medicine Research Center The Third Affiliated Hospital Sun Yat‐sen University Guangzhou China; ^4^ College of Medicine and Forensics Xi'an Jiaotong University Health Science Center Xi'an China; ^5^ Department of Laboratory Medicine First Affiliated Hospital of Gannan Medical University Ganzhou China; ^6^ Shenzhen Key Laboratory of Chinese Medicine Active Substance Screening and Translational Research Shenzhen China

Dear Editor,

Extrachromosomal circular DNA (eccDNA) is a mobile, circular DNA molecule that originates from but exists independently of linear chromosomes.^1^ Its characteristics and potential function in liver failure remain elusive. Herein, we established a reliable workflow for purifying the internal eccDNAs harboured by plasma‐derived extracellular vesicles (EVs) and characterization of these EVs‐eccDNAs in liver failure. Additionally, the impact of liver failure‐specific circulating EVs‐eccDNAs on the hepatocytes was evaluated by synthetic eccDNAs transfection and RNAseq analysis.

This study recruited 22 participants, including 13 patients with liver failure and nine healthy individuals. Detailed information is provided in Table S1 and Material S1. Patients were diagnosed with liver failure using established criteria, and their hepatic function was matched accordingly.^2^, ^3^, ^4^ Subsequently, we isolated plasma‐derived EVs from both healthy control individuals and liver failure.^5^, ^6^ Electron microscopy images showed that both healthy control EVs (HCEVs) and liver failure patient EVs (LFEVs) exhibited the typical “cup‐shaped” morphology with similar average diameters around 100 nm (Figure 1A). Nano‐flow cytometry indicated that the concentration of LFEVs was significantly higher than that of HCEVs, but no significant difference in size was found (Figure 1B). Western blot analysis confirmed small EV markers CD9, CD81 and TSG101 were present, while the negative marker Mitofilin was absent (Figure 1C). Notably, LFEVs had a higher level of CD9 than HCEVs. Our data revealed an increased presence of EVs in the peripheral circulation of liver failure patients.

**FIGURE 1:**
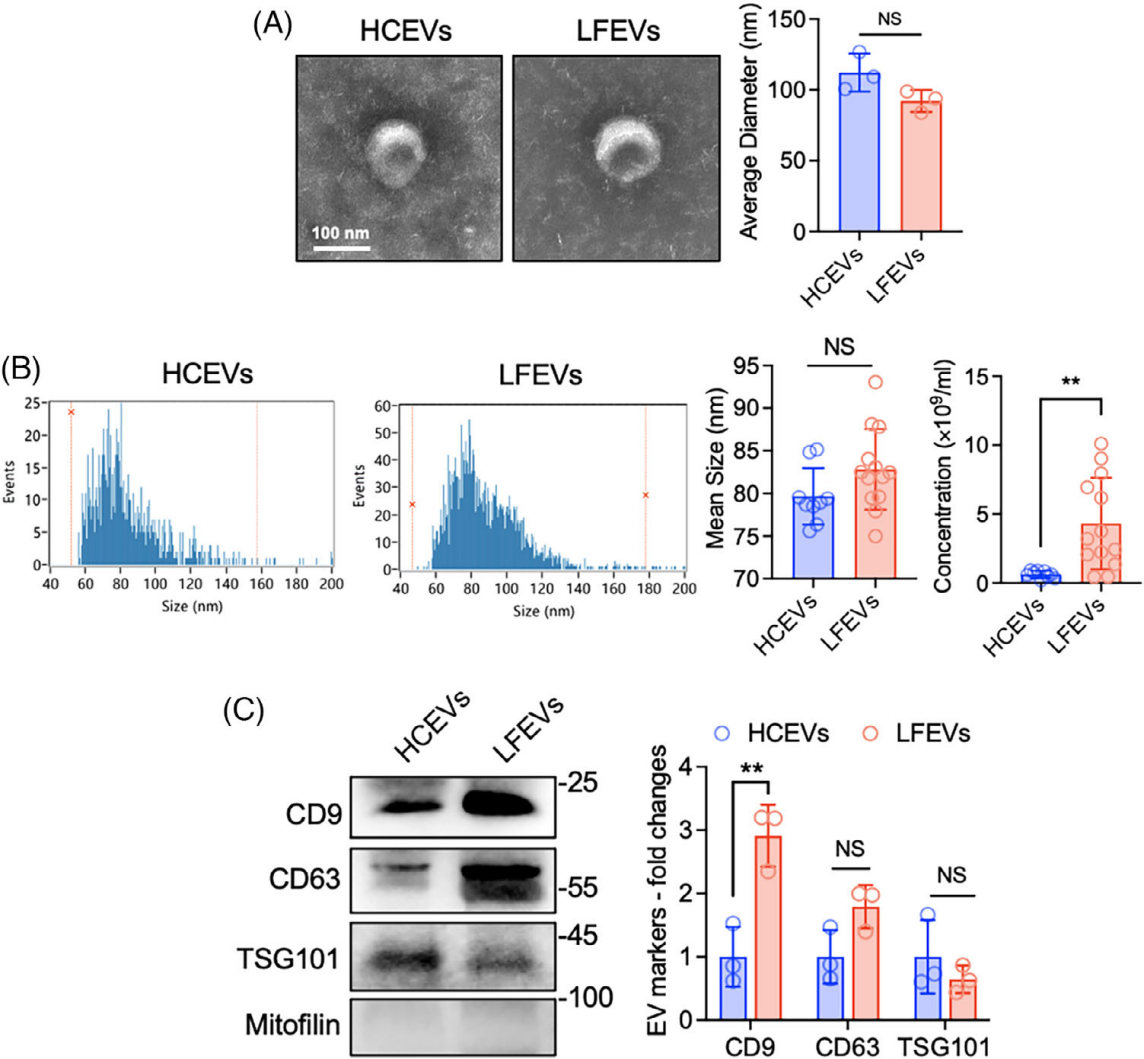
The up‐regulation of plasma extracellular vesicles in liver failure patients. (A–C) Extracellular vesicles (EVs) were isolated from the 500 µL plasma of either healthy control individuals or liver failure patients using a multi‐step ultracentrifugation process. The plasma was centrifuged at 3,000×g for 20 min, followed by centrifugation of the supernatant at 10,000 × *g* for 30 min. The resulting supernatant was diluted with phosphate‐buffered saline (PBS) to a final volume of 30 mL and then centrifuged at 100,000 × g for 3 h. The pellet was washed with PBS, followed by another round of centrifugation at 100,000 × *g* for 3 h to resuspend the final pellet in 100 µL of PBS (Detailed isolation methods can be found in Supporting Information Methods). Aliquots of 5, 10, and 50 µL of HCEVs and LFEVs were used for electron microscopy, nano‐flow cytometry, and Western blot respectively. (A) Electron microscopic images showed the morphology of HCEVs and LFEVs. The average diameters of HCEVs from three healthy individuals and LFEVs from three liver failure patients were obtained through electron microscopy. (*n* = 3, mean ± SD, Student's t‐test, *p < 0.05). (B) The size and concentration of HCEVs and LFEVs were measured using nano‐flow cytometry. (*n* = 9 for HCEVs and 13 for LFEVs, mean ± SD, Student's t‐test, **p < 0.01, NS represented no significant changes). (C) Western blot analysis was performed to examine the presence of small EV (exosome) positive markers, including CD9, CD63, and TSG101, as well as the large EV marker Mitofilin in both HCEVs and LFEVs. (*n* = 3, mean ± SD, Student's t‐test, **p < 0.01, NS represented no significant changes).

We then isolated eccDNA from HCEVs and LFEVs using the process illustrated in Figure 2A. Specifically, eccDNAs with > 75 bp overlap of a certain gene were defined as “eccGenes” in the study.^7^ The read sizes for HCEVs and LFEVs were similar (Figure 2B and Table S2). We then identified that LFEVs had a significantly higher number of eccDNAs compared to HCEVs (Figure 2C and Table S2). The normalized eccDNA count per million mapped reads (EPM) was significantly higher in LFEVs (Figure 2D). Additionally, the GC content and flanking regions of eccDNAs from LFEVs were higher than those from HCEVs (Figure 2E,F). The percentage of EPM across all chromosomes was similar for both LFEVs and HCEVs (Figure 2G, Figure S1A and Material S2). Overall, these data indicate that LFEVs carry a higher abundance of eccDNAs than HCEVs.

**FIGURE 2:**
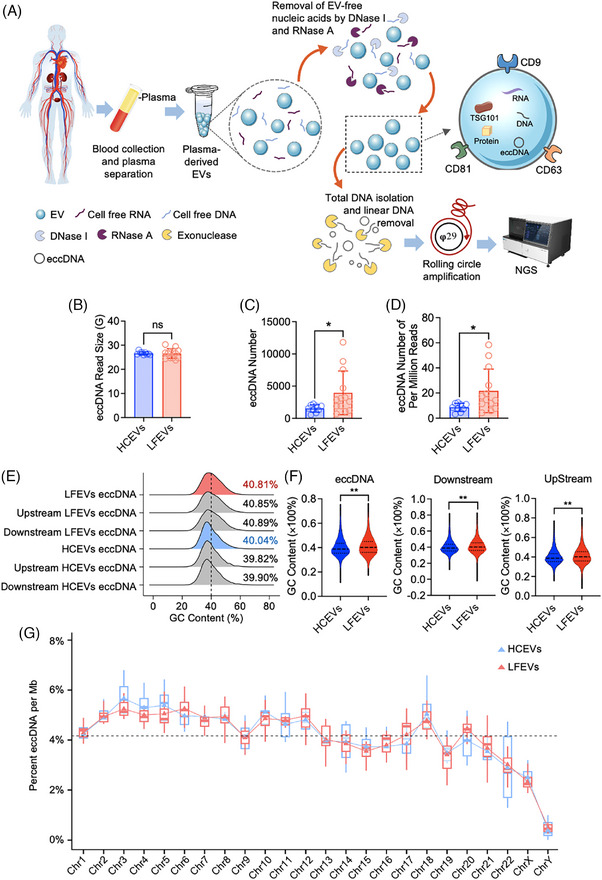
Identification of EVs carried eccDNA in liver failure patients. (A) Workflow of eccDNA purification and sequencing from plasma EVs. Firstly, EVs were isolated from the 0.5 mL plasma of either healthy control individuals or liver failure patients using a multi‐step ultracentrifugation process. The plasma was centrifuged at 3000 × *g* for 20 min, followed by centrifugation of the supernatant at 10 000 × *g* for 30 min. The resulting supernatant was diluted with PBS to a final volume of 30 mL and then centrifuged at 100 000 × *g* for 3 hours. The pellet was washed with PBS, followed by another round of centrifugation at 100 000 × *g* for 3 hours to collect the final pellet. Secondly, the isolated EVs underwent treatment with both DNase I and RNase A to eliminate cell‐free DNA and RNA molecules present outside the EV particles. Thirdly, the total DNA inside the EVs was extracted using a circulating DNA isolation kit, and linear DNA fragments were removed through exonuclease digestion. Finally, the remaining circular DNA molecules in each sample were amplified using rolling circle amplification, and the resulting product was subjected to high‐throughput sequencing (Circle‐seq procedures). For detailed methods of EV isolation and EV‐eccDNA detection, please refer to the Supporting Information file Methods. (B) The high‐throughput sequencing output was compared between LFEVs and HCEVs (Student's *t*‐test, ns = no significant changes). (C, D) A comparison was made between the number of eccDNA in LFEVs and HCEVs, with normalization based on the number of eccDNA per million mapped reads (EPM) (mean ± SD, Student's t‐test, *p < 0.05). (E) The GC content distribution was analyzed for eccDNA, along with the downstream and upstream regions of equivalent length in eccDNAs. (F) The GC content of eccDNA was compared between LFEVs and HCEVs using the Wilcoxon rank‐sum test (mean ± SD, **p < 0.01). (G) The percentages of the number of eccDNAs per Mb were calculated for each chromosome, and the dashed line represented the average across all chromosomes.

We then analyzed the eccDNA lengths in HCEVs and LFEVs. Figure 3A shows five enriched peaks in LFEVs at 370, 566, 751, 946 and 1124 bp, with a noticeably higher density of eccDNAs in LFEVs compared to HCEVs. We calculated the cumulative frequency of HCEVs and LFEVs containing eccDNAs and found that eccDNA lengths in LFEVs were much shorter than those in HCEVs (Figure 3B). Additionally, we found that LFEVs contained a higher ratio of 0.5–1 Kb length eccDNA but a lower ratio of > 2 Kb length eccDNA compared to HCEVs (Figure 3C). Therefore, these findings indicated that LFEVs contained eccDNA with shorter lengths than those in HCEVs. Based on the currently proposed mechanisms of eccDNA formation triggered by genomic stress,^8^ we speculate that this may be related to the increased stress experienced by the genome during the progression of liver failure, which leads to the formation of more and shorter eccDNA into the EVs. Then, we found 75 eccDNAs with common start‐end sites in two groups (Figure 3D). As shown in Figure 3E,F, four eccDNAs were more frequently present in LFEVs than in HCEVs among these common start‐end eccDNAs. Subsequently, we discovered that these four over‐represented LFEVs‐eccDNAs carried specific regulatory genes, transposable elements and candidate cis‐regulatory elements (Figure 3F). These eccDNAs carried the genes ZMIZ1‐AS1 and ZMYM6, which may further influence liver cell functions.

**FIGURE 3:**
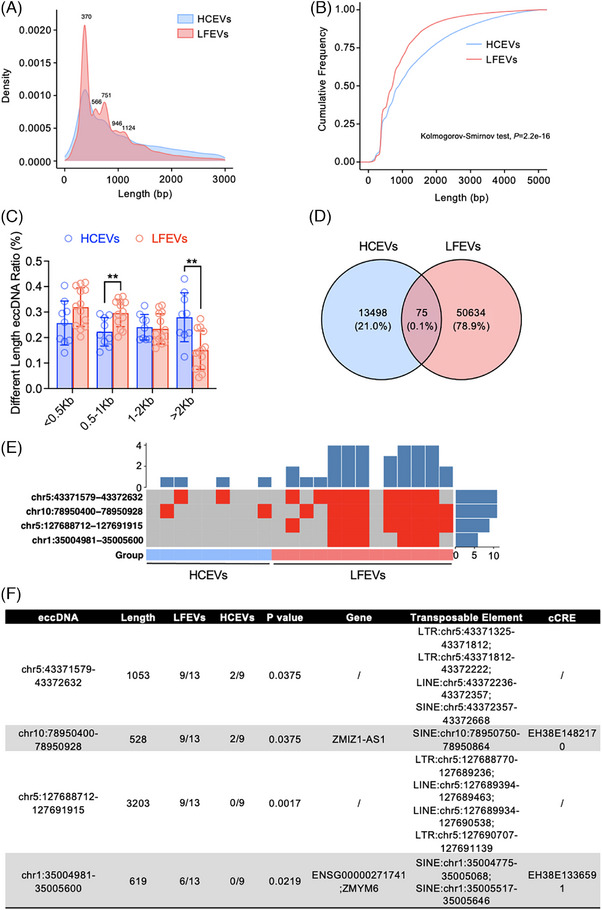
LFEVs contained eccDNA with a shortened length. (A) The density distribution of eccDNA lengths below 3 Kb reveals multiple peaks in both LFEVs and HCEVs. (B) A cumulative frequency plot of eccDNA lengths below 5 Kb was generated for LFEVs and HCEVs, and their distributions were compared using a Kolmogorov‐Smirnov test. (C) A comparison of the ratio of length distribution of eccDNAs between LFEVs and HCEVs was conducted using a Student's t‐test (**p < 0.01). (D) Venn diagram showing the overlap of eccDNAs detected in both LFEVs and HCEVs. (E) Heatmap plot showed the number of significant eccDNAs with the same start‐end between the LFEVs and HCEVs (Wilcoxon rank‐sum test, *p < 0.05). In the heatmap, the presence of eccDNA was indicated by the red colour in each cell. The rows of the heatmap represent different eccDNAs, while the columns represent different groups. (F) Function annotation of the significant eccDNAs, including gene, transposable element, and candidate cis‐regulatory element (cCRE).

Using the LAMA method,^7^ we synthesized artificial eccDNA^[chr10:78950400‐78950928]^ and eccDNA^[chr1:35004981‐35005600]^, referred to as eccZMIZ1‐AS1 and eccZMYM6, respectively (Figure 4A and Tables S3 and S4). Digestion of the linear A fragment with NdeI or SacI produced two lower DNA bands, while artificial eccZMIZ1‐AS1 and eccZMYM6 showed only one higher band, indicating successful synthesis of both (Figure 4B). The synthesized eccZMIZ1‐AS1, eccZMYM6 and a random control eccDNA (eccRandom) were transfected into HepG2 cells via electroporation (Figure S2A), and the cells were collected for genome‐wide messenger RNA (mRNA) sequencing to assess expression changes. The RNAseq results show that 212 mRNAs upregulated and 50 mRNAs downregulated in eccZMIZ1‐AS1 (Figure 4C and Material S3). However, the eccZMYM6 group exhibited no significant changes in mRNA expression (Figure 4D). Subsequently, we analyzed the Kyoto Encyclopedia of Genes and Genomes (KEGG) signalling pathways of differentially expressed genes and found that eccZMIZ1‐AS1 primarily regulated the PI3K‐Akt and HIF‐1 signalling pathways (Figure 4E). Gene Ontology (GO) enrichment analysis showed that eccZMIZ1‐AS1 primarily promotes cellular responses to hypoxia, with regulated genes mainly located in the vesicle membrane and associated with calcium‐dependent phospholipid binding (Figure 4F). Finally, we found that the genes regulated by eccZMIZ1‐AS1 were primarily centred around the cellular processes including response to hypoxia, regulation of lipid metabolic process and exocytic vesicle membrane (Figure 4G). Previous evidence indicated that HIF‐1α mediates acute liver failure induced by LPS/D‐GalN.^9^ Also, previous studies show that lipid metabolism influences intrahepatic macrophage reprogramming, regulating acute‐on‐chronic liver failure caused by the hepatitis B virus.^10^ Currently, no studies have established a direct link between liver failure and the exocytic vesicle membrane. These findings suggest that the liver failure‐specific circulating EVs‐eccDNAs may exert divergent molecular effects on cells, implying that some of them may accelerate the progression of liver failure through systemic impacts on various organs.

**FIGURE 4:**
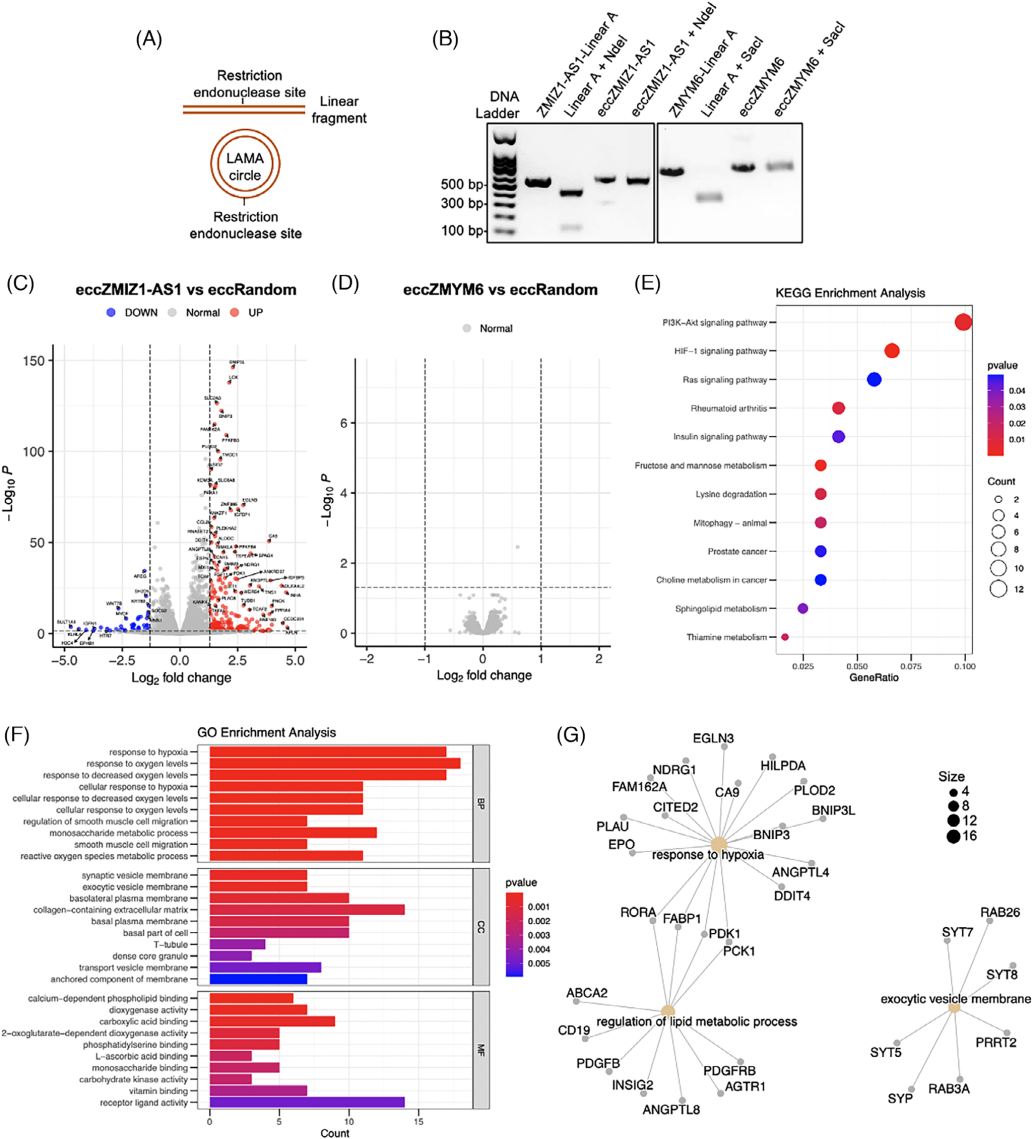
EccZMIZ1‐AS1 regulated the functions and signalling pathways transduction of liver cells. (A) Principle of the identification of artificial eccDNA using single restriction endonuclease digestion. (B) Digestion with NdeI or SacI on the linear A fragment resulted in two lower DNA bands, while artificial eccZMIZ1 or eccZMYM6 showed only one higher band after digestion. (C, D) Volcano plots showed the differential expression of genes following the transfection of synthesized eccZMIZ1‐AS1 and eccZMYM6. The grey dots represented genes that showed normal expression levels without significant changes. The blue dots in the chart represented significantly down‐regulated genes with a log2 fold change < 1.3, while the red dots represented significantly up‐regulated genes with a log2 fold change > 1.3. (E) A dot plot displayed the Kyoto Encyclopedia of Genes and Genomes (KEGG) pathway analysis results for the differentially expressed genes (DEGs) following the transfection of eccZMIZ1‐AS1. (F) A bar plot displayed the Gene Ontology (GO) pathway analysis results for the DEGs. (G) The interaction network of the significant pathway from GO Functional enrichment analysis.

In conclusion, our study presents a reliable method for isolating and characterizing EVs‐eccDNAs, offering insights into their disease‐associated features in liver failure. The activation of specific signalling pathways by eccDNA in HepG2 cells implies its role in liver failure pathogenesis, suggesting its potential as a diagnostic marker and target for therapeutic interventions. However, limitations include the exclusive use of the HepG2 cell line to validate eccZMIZ1‐AS1's functions and the lack of exploration into the specific mechanisms by which it influences liver failure. These aspects require further investigation in future studies.

## AUTHOR CONTRIBUTIONS

Zhigang Li and Xi Xiang planned the project and conceived the experiments. Yongbing Qian, Xiaoning Hong, Yang Yu, Cong Du, Chen Chen, Wenjun Xiao, Jing Li, Jiaying Yu, Tianyu Zhong, and Jiang Li performed the experimental works and analyzed the data. Yongbing Qian, Xiaoning Hong, Yang Yu, Xi Xiang and Zhigang Li conceived the data and wrote the manuscript. All authors approved the final version of the manuscript.

## CONFLICT OF INTEREST STATEMENT

The authors declare no conflict of interest.

## FUNDING INFORMATION

This work was supported by the National Natural Science Foundation of China (32200638 to Z.L.); Basic and Applied Basic Research Fund of Guangdong Province (2021A1515110512 to Z.L., 2023A1515010090 to Z.L. and 2022A1515110137 to Y.Y.); Shenzhen Science and Technology Innovation Program (JCYJ20230807110316034 to Z.L., JCYJ20210324134612035 to Z.L. and JCYJ20220530145014033 to X.X.); Research Start‐up Fund of the Seventh Affiliated Hospital of Sun Yat‐sen University (ZSQYBRJH0021 to Z.L. and 592026 to X.X.); Guangdong Provincial Key Laboratory of Digestive Cancer Research (2021B1212040006 to X.X.); The Open Fund of Guangdong Provincial Key Laboratory of Digestive Cancer Research (GPKLDCR202206M to X.X.); Fundamental Research Funds for the Central Universities of Sun Yat‐sen University (Grant 2023KYPT02 to X.X.).

## ETHICS STATEMENT

All subjects gave their informed consent for inclusion before they participated in the study. The human ethics of this study was approved by Shanghai Jiao Tong University School of Medicine, Renji Hospital Ethics Committee (KY2021‐063‐B).

## Supporting information



Supporting Information

Supporting Information

Supporting Information

Supporting Information

Supporting Information

## Data Availability

The data that support the findings of this study are available from the corresponding author upon reasonable request. The raw data of the eccDNA sequencing and RNAseq experiments have been deposited into the NCBI Sequence Read Archive (SRA) with the accession number PRJNA1002003.
