# Supplementary material for: Characterization and functional analysis of extrachromosomal circular DNA discovered from circulating extracellular vesicles in liver failure
Source: Clin Transl Med. 2024 Oct 15;14(10):e70059. doi: 10.1002/ctm2.70059 (PMC11479749; doi:10.1002/ctm2.70059)

**Supplemantray Figures and Tables**


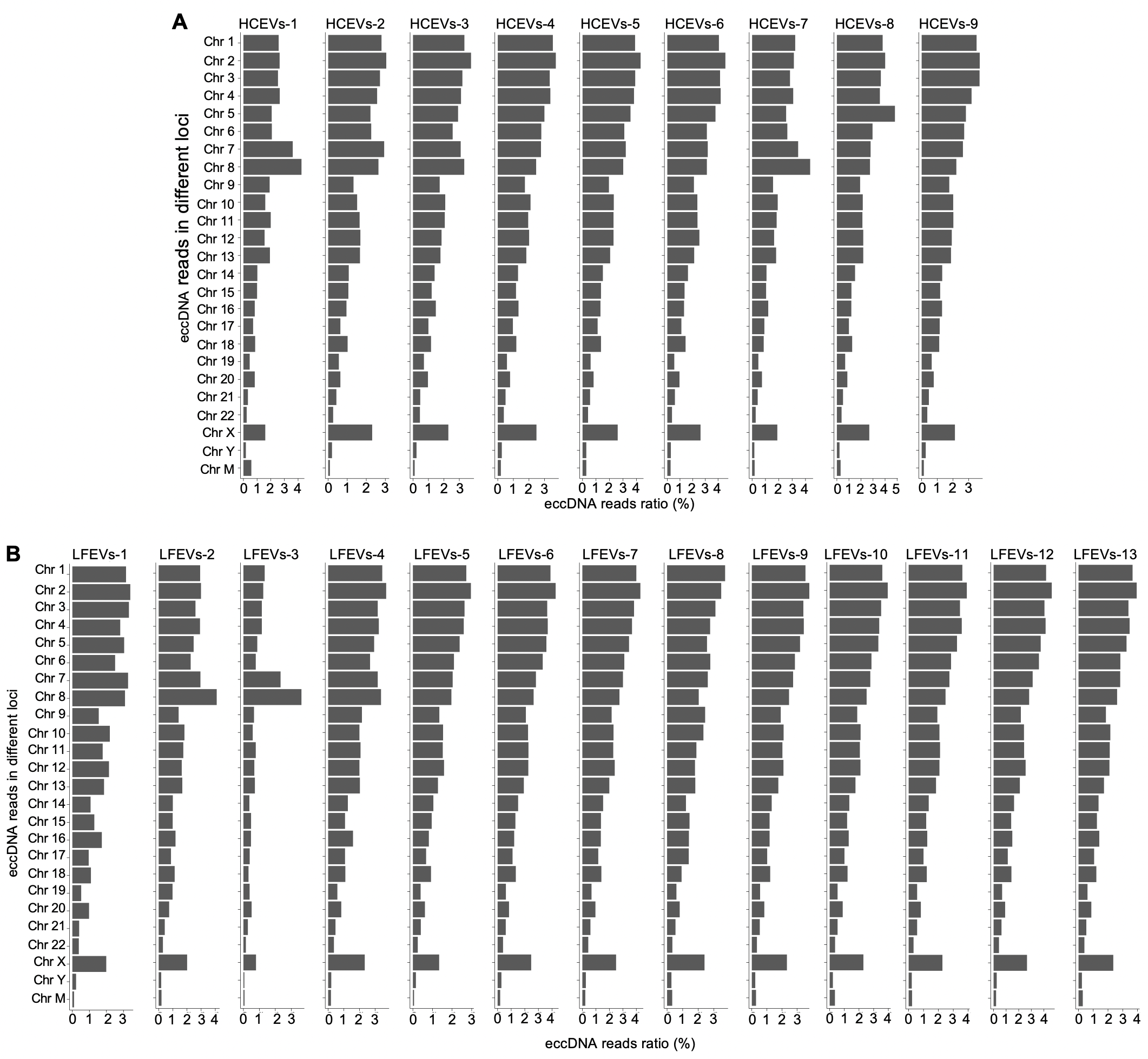


**Figure S1. The ratio of EPM in all chromosome of HCEVs and LFEVs.** (A-B) Bar plot displaying the ratio of eccDNA mapped reads on each individual chromosome among the HCEVs (A) and LFEVs (B).


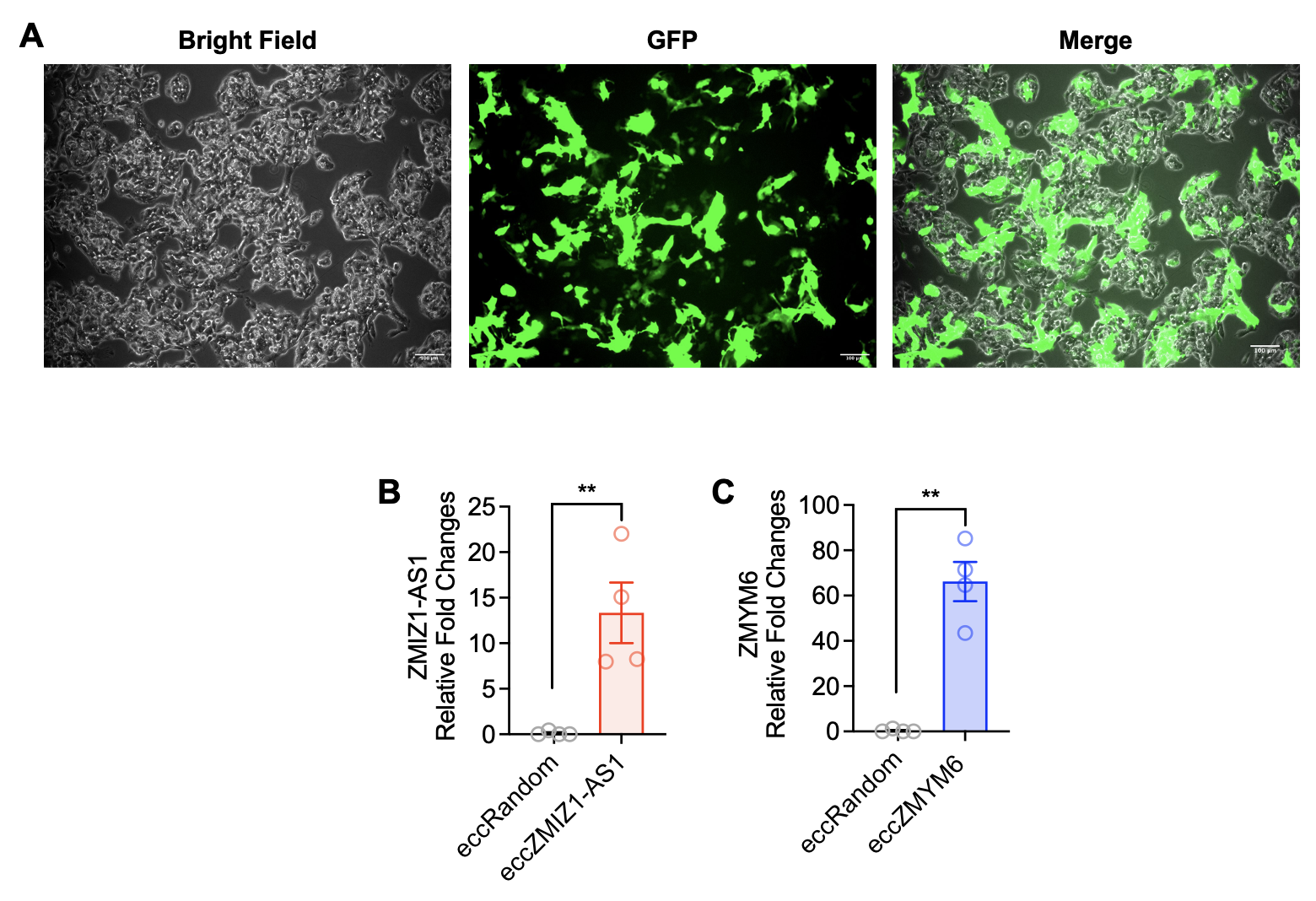


**Figure S2. Transfection of eccZMIZ1-AS1 and eccZMYM6 into HepG2 cells.** (A) HepG2 cells were electroporated with GFP plasmids using the same transfection protocol. After 48 hours of transfection, the expression level of GFP was detected by fluorescence microscopy.

**Table S1. Informations of patients with Liver Failure vs health control.**

**
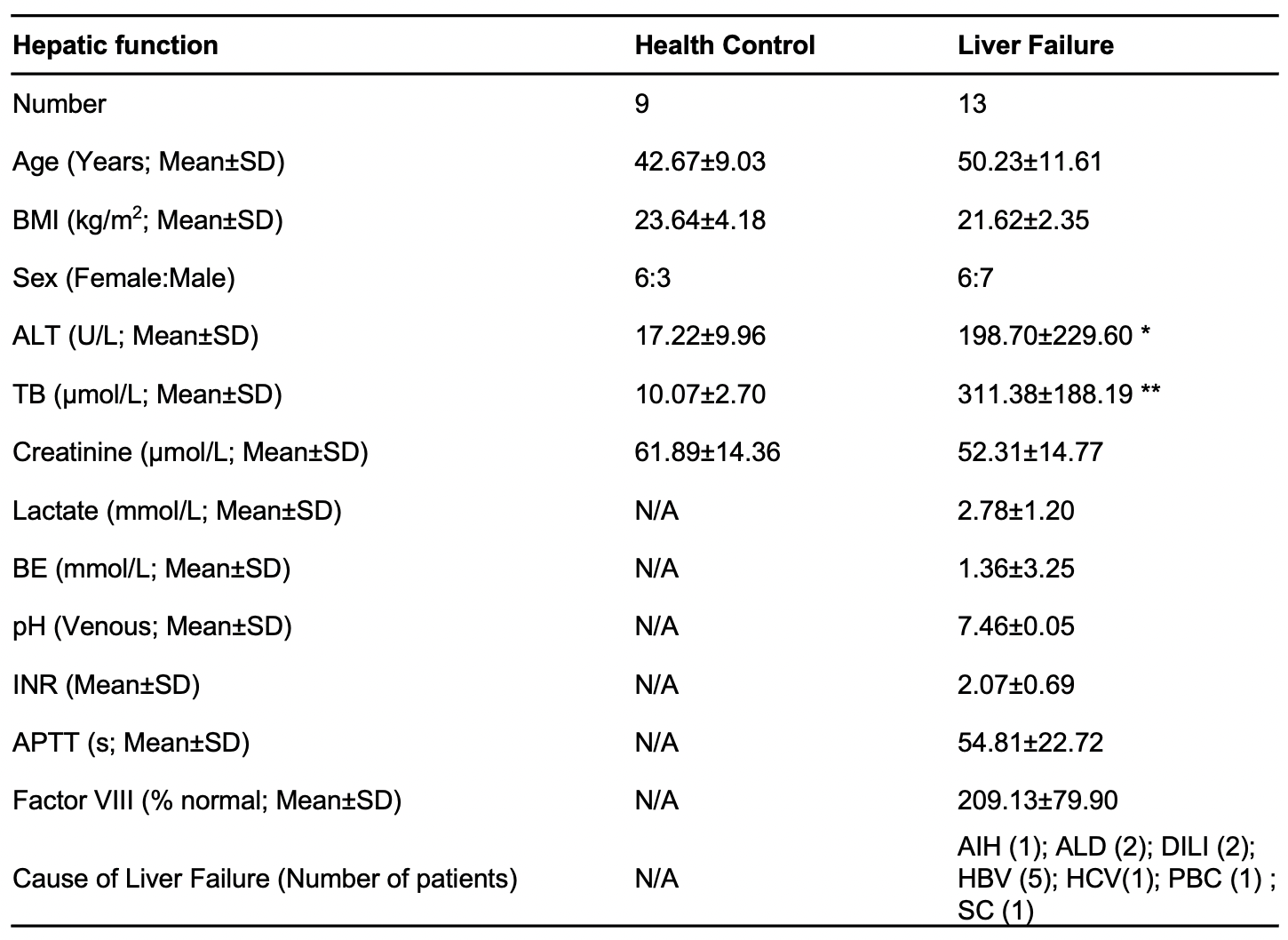
**

**AIH**, Autoimmune hepatitis; **ALD**, Alcoholic liver disease; **DILI**, Drug induced liver injury; **HBV**, Hepatitis B virus; **HCV**, Hepatitis C virus; **PBC**, Primary biliary cholangitis; **SC**, Schistosomiasis cirrhosis; **N/A**, not applicable. Data were analyzed with unpaired t-test (**p* < 0.05; ***p* < 0.01).

**Table S2. Sequencing data of eccDNA contained in HCEVs and LFEVs**


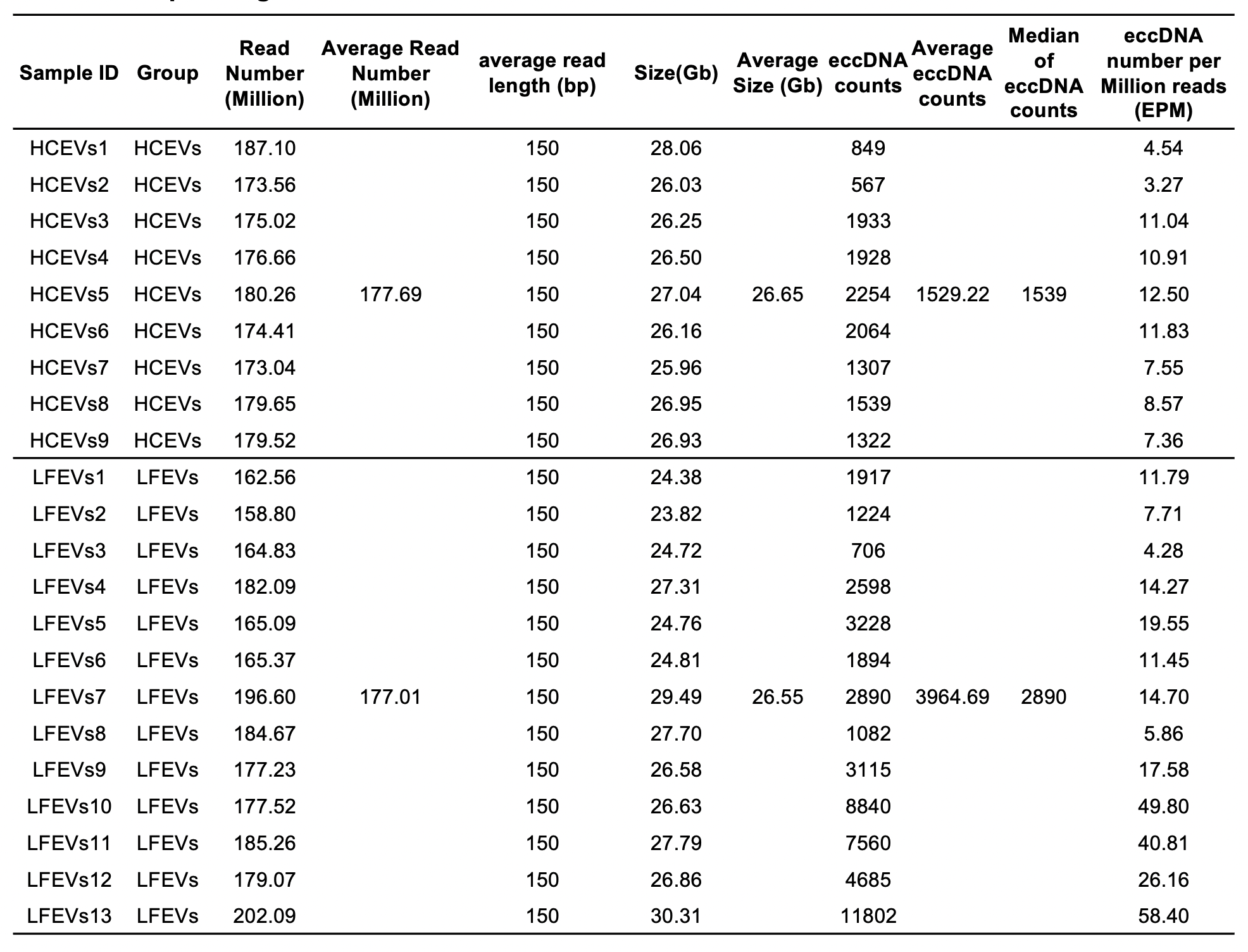


**Table S3. Information of eccZMIZ1, eccZMYM6 and eccRandom**


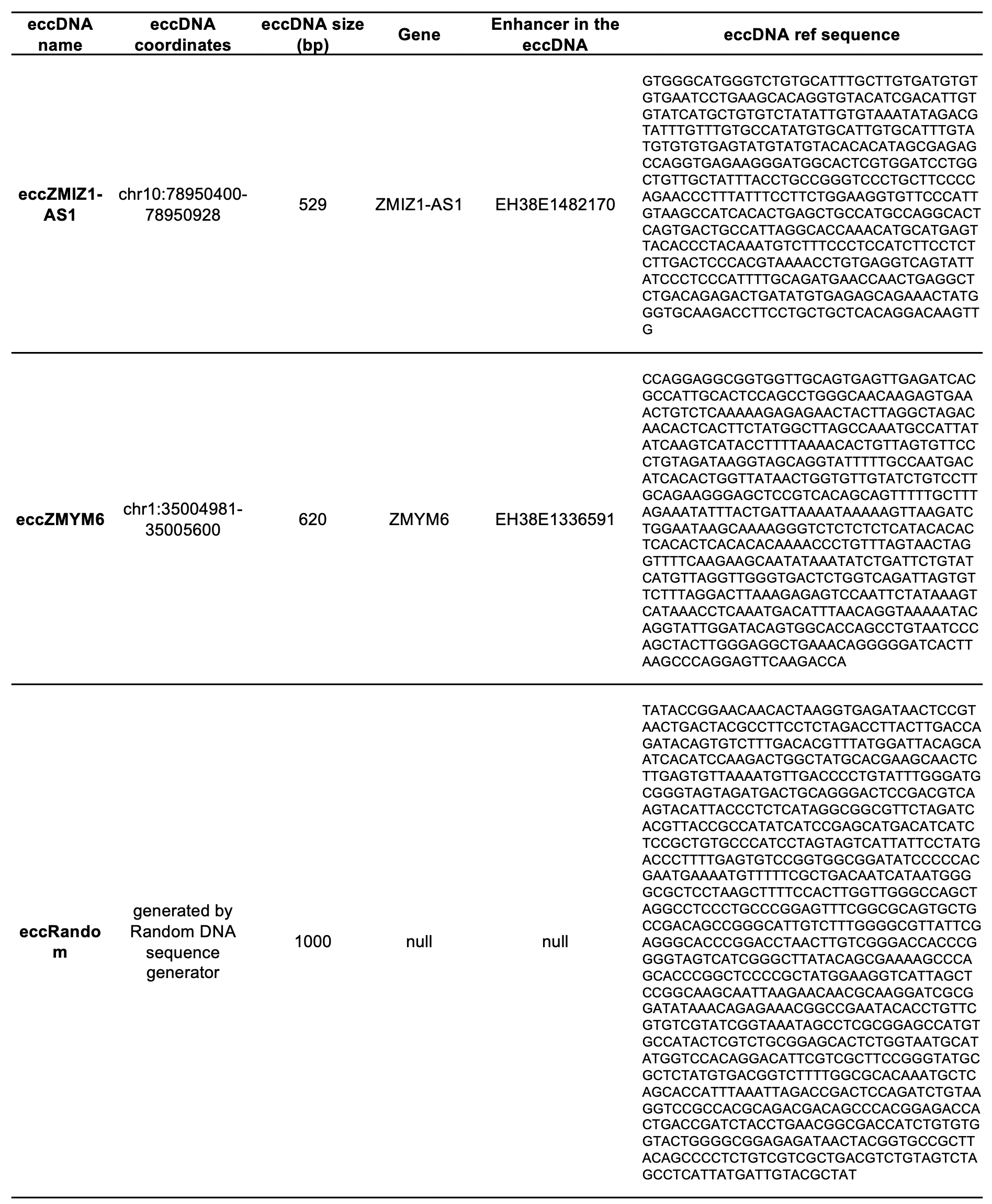


**Table S4. Synthetic linear A and linear B DNA fragments and primers for LAMA reaction**


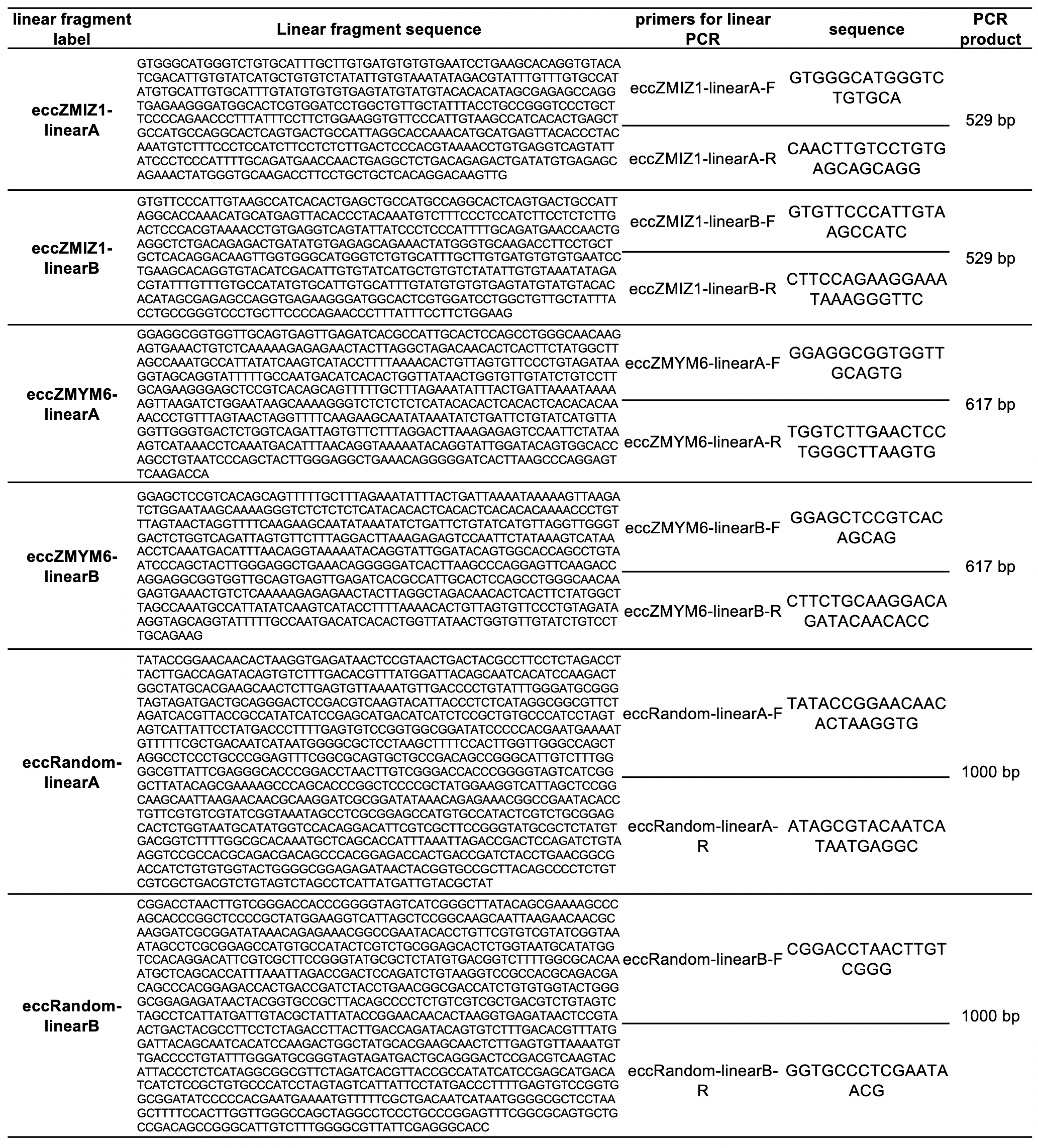

Supplement: Supplementary file 1 — Supporting Information [file CTM2-14-e70059-s001.docx]
