# Supplementary material for: Characterization and functional analysis of extrachromosomal circular DNA discovered from circulating extracellular vesicles in liver failure
Source: Clin Transl Med. 2024 Oct 15;14(10):e70059. doi: 10.1002/ctm2.70059 (PMC11479749; doi:10.1002/ctm2.70059)
Supplement: Supplementary file 5 — Supporting Information [file CTM2-14-e70059-s005.docx]

**Materials and Methods**

**Case recruitment**

All inpatients with liver failure were from Shanghai Renji Hospital, Shanghai Jiaotong University. The patients with liver failure were chosen randomly and had to meet specific criteria: 1) Acute liver failure: Defined as the emergence of acute liver damage leading to coagulopathy and/or encephalopathy in the absence of pre-existing liver disease [1]; 2) Acute on chronic liver failure: Characterized by a sudden decline in liver function in patients with previously diagnosed or undiagnosed liver conditions, accompanied by multiorgan failure according to APSAL criteria [2]; 3) Decompensated chronic liver failure: This is defined as the occurrence of esophageal varices, hepatic encephalopathy, or ascites in patients who already have established liver cirrhosis [3]. Both patients and healthy individuals participating in the study were given informed consent. And the human ethic of this study was approved by Shanghai Jiao Tong University School of Medicine, Renji Hospital Ethics Committee (KY2021-063-B).

**Plasma extracellular vesicles isolation**

Accroding to the previously established protocol, extracellular vesicles (EVs) were isolated from the plasma of both healthy individuals and liver failure patients [4, 5]. First, the 0.5ml plasma was diluted with 33 ml of PBS and subsequently subjected to centrifugation at 3000×g for 20 minutes at 4°C to remove any cellular debris. The resulting supernatant was then collected and subjected to further ultra-centrifugation at 10,000×g for 30 minutes at 4°C to eliminate any microvesicles and apoptotic bodies. The resulting supernatant was diluted with PBS to a final volume of 30 ml and ultracentrifuged at 100,000×g for 3 hours. The pellet was then washed with PBS, followed by another round of ultracentrifugation at 100,000×g for 3 hours to collect the final pellet. Finally, the EV pellets were promptly resuspended in 100 µl PBS and utilized for further experimentation.

**Transmission electron microscopy**

The extracted EVs are first dissolved in a 50-100 µl solution of 2% paraformaldehyde and can be stored at 4°C for one week. Then, 5-10 µl of the EVs solution is added onto a Formvar-carbon coated copper grid. The copper grid (Formvar side facing down) is washed in 100 µl of PBS by placing it onto a droplet of PBS using tweezers. It's important to keep the Formvar side wet and the other side dry throughout all steps. Next, the copper grid is placed onto a 50 µl droplet of 1% glutaraldehyde for 5 minutes, followed by washing in 100 µl of ddH_2_O for 2 minutes (repeat 8 times). The copper grid is then placed onto a 50 µl droplet of uranyl oxalate solution (pH 7.0) for 5 minutes, and then onto a 50 µl droplet of methylcellulose solution for 10 minutes, while working on ice. The copper grid is then placed onto a stainless-steel loop at the top of the sample stage and excess liquid is removed on filter paper. After air drying for 5-10 minutes, the copper grid is placed in a sample box and electron microscope images are taken under 80kV.

**Western blot**

The EVs were ruptured using RIPA buffer (25 mM Tris-Cl, pH 7.4, 150 mM NaCl, 1 mM EDTA, 5% glycerol, and 1% NP-40), consisting of protease inhibitor cocktail and PMSF. The EVs' membrane was disrupted by sonication, and the protein concentrations were determined using the BCA protein assay kit. Samples containing the same amount of protein were loaded onto a 10% SDS-PAGE gel, separated, and then transferred from the gel onto a PVDF membrane using a transfer buffer for electrotransfer. The PVDF membrane was blocked for 1 h at room temperature with 5% non-fat milk, followed by incubation at 4°C overnight with primary antibodies including CD9 (Santa Cruz Biotechnology, sc-13118), CD63 (Santa Cruz Biotechnology, sc-5275), TSG101 (Santa Cruz Biotechnology, sc-7964), and Mitofilin (Proteintech, 10179-1-AP). The membrane was rinsed in TBST (TBS containing 0.1% Tween-20) and incubated with appropriate HRP-conjugated secondary antibodies for 1 h. The protein bands were visualized using the ChemiDoc Imaging System from Bio-Rad and quantified using ImageJ version 1.50i.

**Nano Flowcytometry**

Prior to conducting each experiment, the Flow NanoAnalyzer underwent alignment using polystyrene QC beads from NanoFCM Inc. Afterward, standard nanospheres from the same manufacturer were measured to calibrate the instrument for EV analysis. Once the NanoAnalyzer was both aligned and calibrated, EV samples were diluted in Dulbecco’s PBS (DPBS) to a concentration of 1×10^8^ particles/mL for optimal measurement. To avoid detecting particle swarms, samples and blanks (200-800 events) were measured for one minute using a laser power of 15 mW as an excitation source, constant pressure of 1 kPa, and at an event rate between 2500 to 12,000 events/min as recommended by the manufacturer [6]. The distribution of size and concentration of plasma EVs samples was then detected by the NanoAnalyzer. Finally, the mean size and concentration of EVs were calculated using the NanoAnalyzer software.

**EccDNA purification from EVs and high-throughput sequencing**

The procedure for EV-eccDNA purification and sequencing is illustrated in Figure 2A. First, 100 µL EV per sample was treated with DNase I and RNase A to remove the cell-free DNA and RNA outside the EV particles. Briefly, each 120 µL reaction consisted of 100 µL EV, 4 µL DNase I (ThermoFisher, 1 unit/µL), 4 µL RNase A (ThermoFisher, 10 ug/µL) and 12 µL 10 × reaction buffer with MgCl_2_ (ThermoFisher). Then the reaction was incubated at 37 ℃ for 1 h for sufficient digestion of the DNA and RNA outside EV particles. Subsequently, the digestion was stopped by mixing with 10 µL 50mM EDTA solution and heating at 65 ℃ for 10 min; Second, the total DNA in EV sample was isolated using VAHTS Serum/Plasma Circulating DNA Kit (Vazyme), according to the manufacturer’s protocol . The final EV-derived DNA was eluted in 80 µL nuclease-free water and stored in -20℃ or -80℃ freezer for subsequent use. Third, the EV-eccDNA was purified, sequenced and deciphered by Circle-seq, which was described previously [7-9]. The total EV-derived DNA was digested with Plasmid-Safe ATP-dependent DNase (PSD) (Epicenter) in a 50 µL reaction system containing 40 µL EV-derived DNA, 5 µL 10 × PSDreaction buffer, 2 µL ATP, 2 µL PSD (10 U/µL) and 1 µL ddH_2_O, at 37 ℃ for 48 h. Then purify the remaining circular DNA by WAHTS^®^ DNA clean beads (Vazyme) and perform rolling circle amplification (RCA) using phi29 polymerase (ThermoFisher) at 30 ℃ continuously for 48 h according to the manufacturer’s instruction. All the RCA products were purified again using DNA clean beads (Vazyme) and sent for next-generation-sequencing (NGS) in BGI company.

**EccDNA Identification from Circle-seq data**

The high-throughput sequencing reads were aligned to the human reference genome (GRCh38) using BWA-MEM(PMID: 35253835). The Circle-Map (v1.1.4) software (<https://github.com/iprada/Circle-Map>) was used to detect the coordinates of eccDNA from sorted BAM files [10]. In order to enhance the accuracy of eccDNA detection, multiple filtering steps were applied with the following specific settings: (1) Split reads ≥ 2, (2) Circle score ≥ 200, (3) Coverage continuity ≤ 0.9, and (4) The standard deviation of coverage is smaller than the mean coverage across the entire eccDNA region.

**Genomic annotation of eccDNA**

The genome features were obtained from GENCODE version 41. Additional annotation data for various genomic elements, including 3'UTR, 5'UTR, CpG island, exon, Gene2KbD, Gene2KbU, and intron, were downloaded from the UCSC table browser (<https://genome.ucsc.edu/cgi-bin/hgTables/>). Additionally, data for repetitive DNA annotation was obtained from RepeatMasker open-4.0.5 (<http://repeatmasker.org>). To quantify the quantity of eccDNA mapped to specific elements [11], we utilized BedTools multicov and BedTools groupby. The normalized mapping ratio was computed by dividing the percentage of a specific element by its percentage in the nuclear genome.

**Differential eccDNA Analysis**

The Wilcoxon rank-sum test was employed to identify differential eccDNA with the same start-end site. EccDNA showing an absolute log2 fold change > 1.5 and a significance level of *p* < 0.05 were considered statistically significant. To ascertain the quantity of eccDNA within individual genes, we employed BedTools for our analysis. For the purpose of quantification, the relative abundance of eccDNA across each gene was determined using the metric of Transcripts Per kilobase of gene model per Million mapped eccDNA (TPM). To pinpoint genes exhibiting varied eccDNA levels between Liver Failure Extracellular Vesicles (LFEVs) and corresponding Healthy Control Extracellular Vesicles (HCEVs), we conducted an additional Wilcoxon rank-sum test. Genes displaying an absolute log2 fold change > 1.5 and a significance level of *p* < 0.05 were considered statistically significant.

**Artificial eccDNA synthesis and identification**

In this study, to evaluate the intracellular function of LF over-represented eccDNAs, we synthesized three artificial eccDNAs, including eccRandom (1000 bp), eccZMIZ1-AS1^[chr10: 78950400-78950928]^ and eccZMYM6^[chr1: 35004981-35005600]^, by ligase-assisted minicircle accumulation (LAMA) strategy as previously described [12]. The eccRandom acted as a control eccDNA and its sequence was generated by the webtool of “Random DNA sequence generator” (<http://www.faculty.ucr.edu/~mmaduro/random.htm>) with 1000 bp in length and 50% GC-content. Reference DNA sequence of the three eccDNAs is displayed in Supplementary Table S3. For synthesis of one certain eccDNA by LAMA reaction, it requires two linear DNA fragments which were complementary to each other in a “inverse and half-way” manner [12]. Therefore, we synthesized the two linear DNA fragments (termed “linear A” and “linear B” in this study) for each of the three eccDNAs in GenScript company. The reference DNA sequence and PCR primers for amplification of these synthetic linear DNA fragments were listed in Supplementary Table S4. For LAMA reaction, linear A and linear B fragments were amplified from the plasmid containing the synthetic DNA segments using standard PCR. Then equal amounts of purified linear A and B PCR products were mixed with the Taq DNA ligase (NEB) and its buffer. The reaction was performed by 10 cycles of denaturation, annealing and ligation. The LAMA product was then treated with PSD at 37 ℃ for overnight to remove the residual linear DNA. Verification of the circular structure and purity of the artificial eccDNA was conducted by appropriate single restriction endonuclease (RE) digestion.

**Artificial eccDNA nucleofection and RNA sequencing**

To prepare the mixture for nucleofection, 500 ng eccDNA (no more than 5 μL in volume) was mixed with 16.4 μL Nucleofector SolutionTM and 3.6 μL Supplement and incubated at room temperature for about 10 min according to the instruction of Amaxa 4D-Nucleofector X Kit TM (Lonza, #V4XP-3032). HepG2, the liver hepatocellular carcinoma-derived cells, was harvested by 0.25% Trypsin and centrifugated to remove the supernatant. 1×10^6^ HepG2 Cells pellet was resuspended with the mixture above and added to the Nucleocuvette^TM^ Vessel carefully. Nucleofection reaction was conducted by the program of EM110 in a 4D-Nucleofector X Unit (Lonza). After the run completion, cells were transferred to a six-well plate and cultured for 48 hours. Total RNA extraction was performed using Trizol reagent kit (Invitrogen, Carlsbad, CA, USA) according to the manufacturer’s suggestions. RNA quality was evaluated by the Agilent 2100 Bioanalyzer (Agilent Technologies, Palo Alto, CA, USA) and then total mRNA was enriched by Oligo(dT) beads. Then the enriched mRNA was sheared into short fragments using fragmentation buffer and reversely transcribed into cDNA by Next Ultra RNA Library Prep Kit for Illumina (NEB #7530, New England Biolabs, Ipswich, MA, USA). CDNA libraries were sequenced on Illumina Novaseq6000 platform by Gene Denovo Biotechnology Co. (Guangzhou, China).

**RNA-Seq Data Analysis**

Adapters or low-sequencing data were trimmed using fastp (v0.19.6) [13]. Processed clean read pairs were aligned to the reference genome using HISAT (v2.2.4) with default parameters [14]. FeatureCounts (subread v2.0.1) was employed to measure the mapped read counts per gene. The R package “DESeq2” was employed to normalize the raw read counts and identify differential expression [15]. The genes with absolute log2 fold change > 1.3 and FDR < 0.05 were considered statistically significant. GO (Gene Ontology) and KEGG (Kyoto Encyclopedia of Genes and Genomes) enrichment analysis of the differential expression genes were conducted using R package “clusterProfiler” [16].

**Statistical Analysis**

The data analysis involved several statistical tests, including the Wilcoxon rank-sum test, Kolmogorov-Smirnov test, and Student’s t-test. The R version 4.2.0 was utilized for all statistical analyses. Two-sided tests were employed, and a significance level of *p* < 0.05 was considered as indicating statistical significance. Detailed descriptions of these tests can be found in the main text and figure legends.

**Reference**

1. Wendon, J., et al., *EASL Clinical Practical Guidelines on the management of acute (fulminant) liver failure.* Journal of Hepatology, 2017. **66**(5): p. 1047-1081.

2. Sarin, S.K., et al., *Acute-on-chronic liver failure: consensus recommendations of the Asian Pacific association for the study of the liver (APASL): an update.* Hepatology International, 2019. **13**(4): p. 353-390.

3. Olson, J.C., *Acute-on-chronic and Decompensated Chronic Liver Failure: Definitions, Epidemiology, and Prognostication.* Crit Care Clin, 2016. **32**(3): p. 301-9.

4. Chaudhuri, A.D., et al., *TNFalpha and IL-1beta modify the miRNA cargo of astrocyte shed extracellular vesicles to regulate neurotrophic signaling in neurons.* Cell Death Dis, 2018. **9**(3): p. 363.

5. Li, Z., et al., *Astrocytes deliver CK1 to neurons via extracellular vesicles in response to inflammation promoting the translation and amyloidogenic processing of APP.* J Extracell Vesicles, 2020. **10**(2): p. e12035.

6. Yuana, Y., et al., *Atomic force microscopy: a novel approach to the detection of nanosized blood microparticles.* J Thromb Haemost, 2010. **8**(2): p. 315-23.

7. Jiang, X., et al., *Genome-wide characterization of extrachromosomal circular DNA in gastric cancer and its potential role in carcinogenesis and cancer progression.* Cell Mol Life Sci, 2023. **80**(7): p. 191.

8. Moller, H.D., et al., *Circular DNA elements of chromosomal origin are common in healthy human somatic tissue.* Nat Commun, 2018. **9**(1): p. 1069.

9. Sin, S.T.K., et al., *Identification and characterization of extrachromosomal circular DNA in maternal plasma.* Proc Natl Acad Sci U S A, 2020. **117**(3): p. 1658-1665.

10. Prada-Luengo, I., et al., *Sensitive detection of circular DNAs at single-nucleotide resolution using guided realignment of partially aligned reads.* BMC Bioinformatics, 2019. **20**(1): p. 663.

11. Quinlan, A.R., *BEDTools: The Swiss-Army Tool for Genome Feature Analysis.* Curr Protoc Bioinformatics, 2014. **47**: p. 11 12 1-34.

12. Paulsen, T., et al., *Small extrachromosomal circular DNAs, microDNA, produce short regulatory RNAs that suppress gene expression independent of canonical promoters.* Nucleic Acids Res, 2019. **47**(9): p. 4586-4596.

13. Chen, S., et al., *fastp: an ultra-fast all-in-one FASTQ preprocessor.* Bioinformatics, 2018. **34**(17): p. i884-i890.

14. Kim, D., B. Langmead, and S.L. Salzberg, *HISAT: a fast spliced aligner with low memory requirements.* Nat Methods, 2015. **12**(4): p. 357-60.

15. Love, M.I., W. Huber, and S. Anders, *Moderated estimation of fold change and dispersion for RNA-seq data with DESeq2.* Genome Biol, 2014. **15**(12): p. 550.

16. Wu, T., et al., *clusterProfiler 4.0: A universal enrichment tool for interpreting omics data.* Innovation (Camb), 2021. **2**(3): p. 100141.
